# Supplementary material for: HAK-actin, a U-ExM-compatible probe to image the actin cytoskeleton
Source: Cell Rep Methods. 2026 Apr 16;6(5):101408. doi: 10.1016/j.crmeth.2026.101408 (PMC13198096; doi:10.1016/j.crmeth.2026.101408)
Supplement: Document S1. Figures S1–S4 [file mmc1.pdf]

**Cell Reports Methods, Volume *6***

## Supplemental information

# HAK-actin, a U-ExM-compatible probe to image the actin cytoskeleton

**Olivier Mercey, Luc Reymond, Florent Lemaître, Isabelle Mean, Marine H. Laporte, Marine Olivetta, Karin Sadoul, Omaya Dudin, Virginie Hamel, and Paul Guichard**

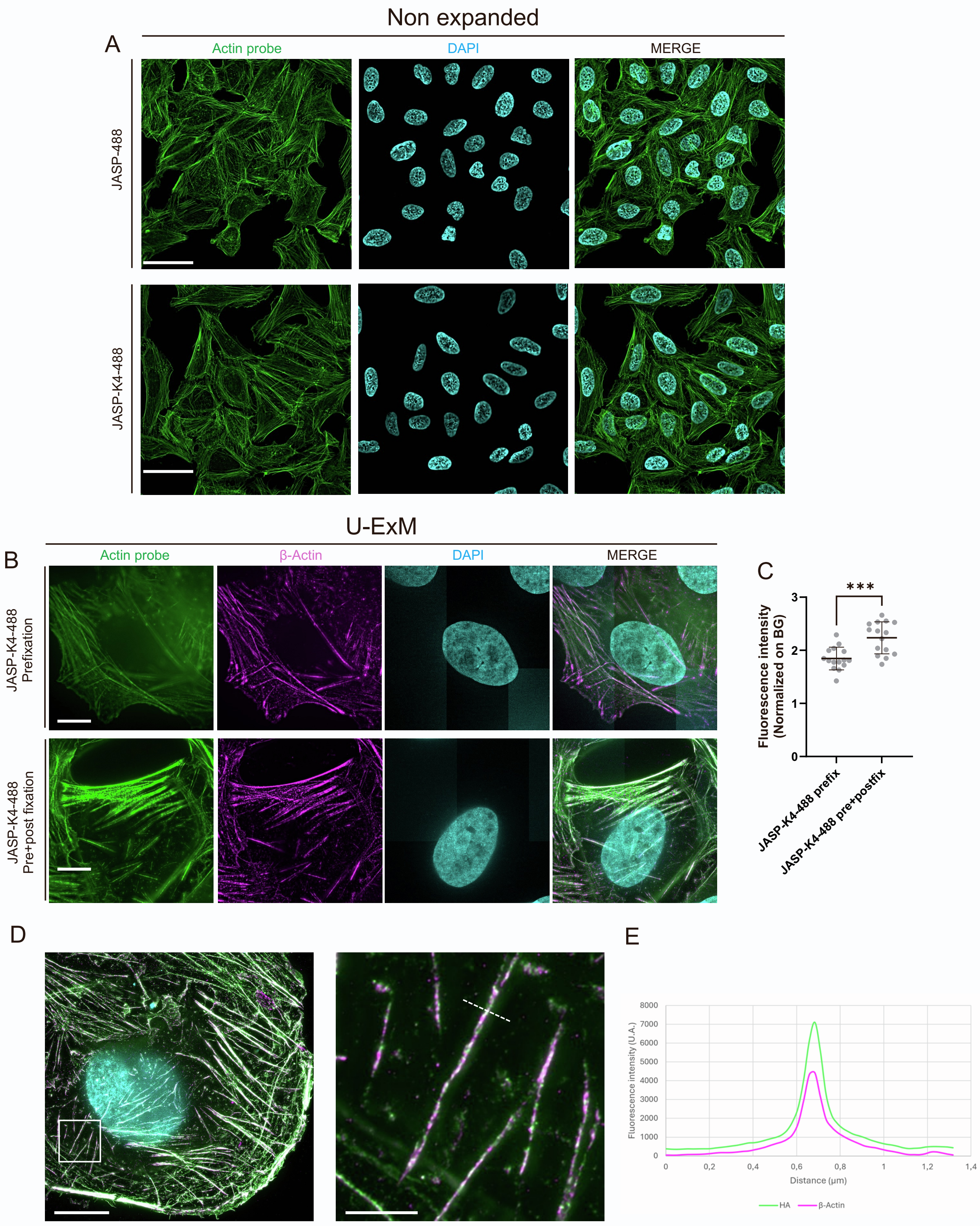


**Supplementary Figure 1. Additional tests to evaluate the actin probes under different conditions, related to Figure 1.**

**(A)** Immunofluorescence images of non-expanded U2OS cells treated with either JASP-488 or JASP-K4-488 (green) together with DAPI (cyan). Scale bar: 50 µm. **(B)** Widefield images of expanded U2OS treated with JASP-K4-488 (green) with or without post-fixation step (PFA 4%, Glutaraldehyde 0,0125%). Scale bar: 10 µm **(C)** Signal intensity measurement of JASP-K4-488 assessing the effect of post fixation (n=15 cells; N=3 independent experiments per conditions). ***p < 0.001, by Mann-Whitney test. **(D)** Example of the overlap between the HAK-actin probe signal and beta actin antibody signal. Scale bar: large image: 10 µm; inset: 2µm. Of note, the image used is the same used in Figure 1E with an inset in a distinct area. **(E)** Plot profile resulting from the dashed line in (D).

PHALLO-K4-HA

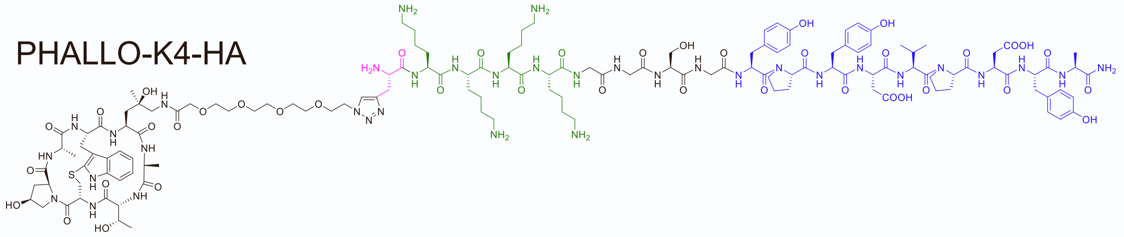

JASP-DAP4-HA

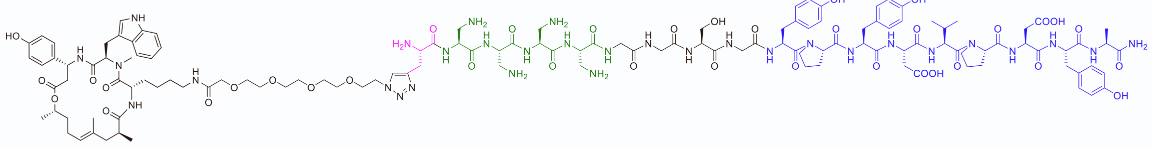

JASP-ORN4-HA

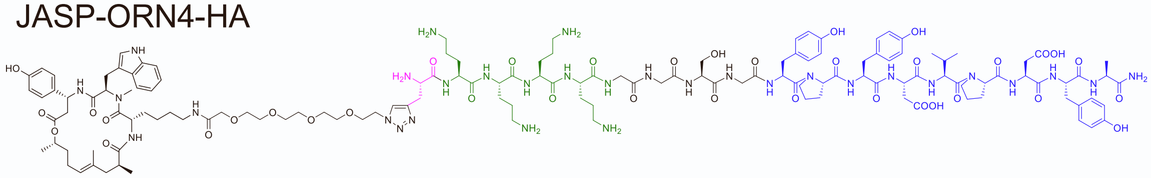

JASP-AF4-HA

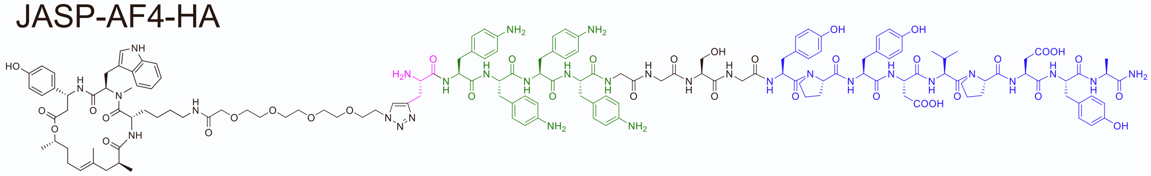

**Supplementary Figure 2. Chemical structure of HAK probes PHALLO-K4-HA, JASP-DAP4-HA, JASP-ORN4-HA and JASP-AF4-HA, related to Figure 2.**

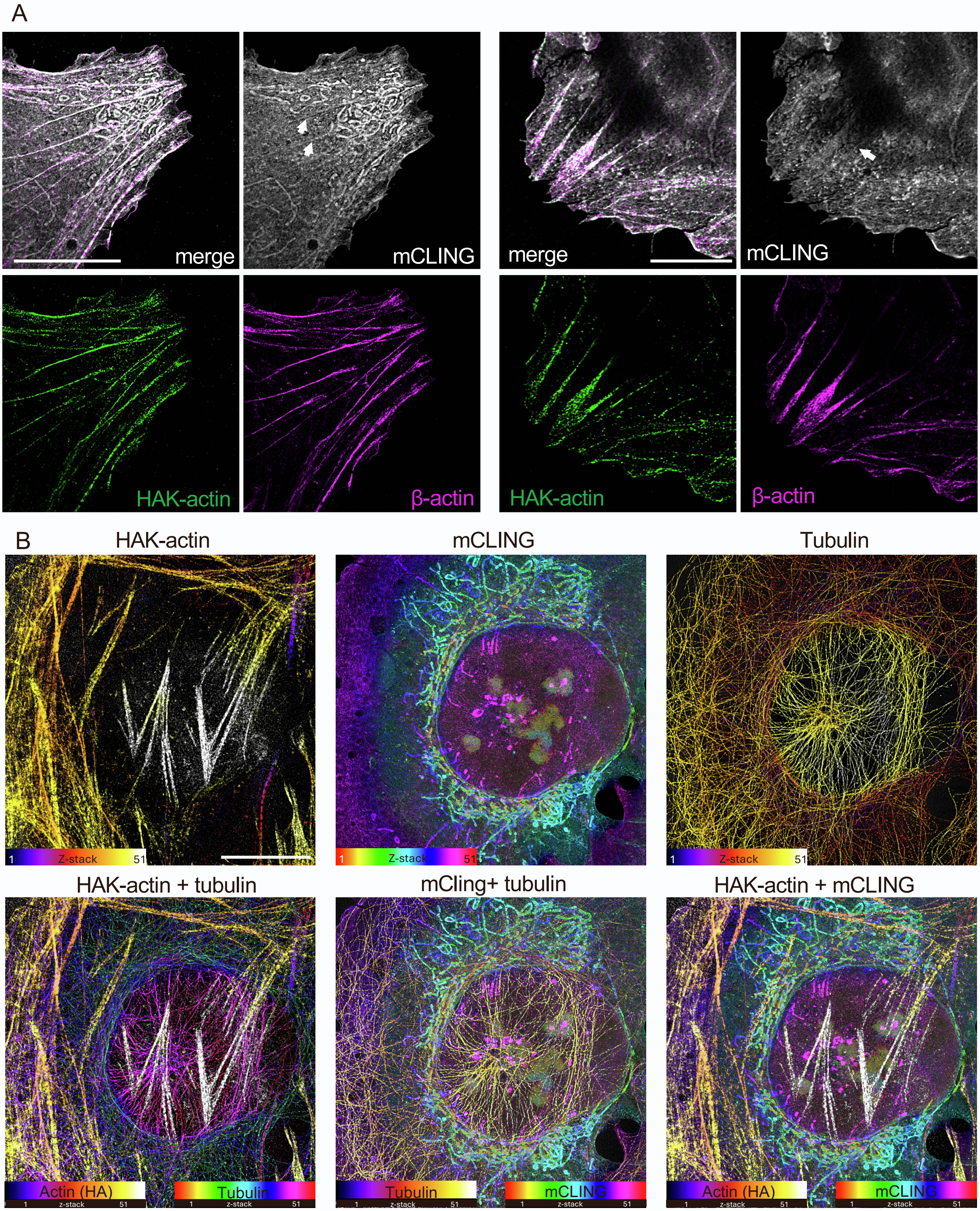


**Supplementary Figure 3. Simultaneous visualization of cellular compartments and cytoskeleton networks, related to Figure 3.**
**(A)** Confocal images of cryo-fixed and expanded U2OS cells stained with HAK-actin (anti-HA, green), Beta-actin (magenta), and membranes (mCLING, Gray). Membrane deformation/groove caused by actin bundles are observable on the mCling channel (white arrows). Scale bars: 10 µm. **(B)** Confocal images of cryo-fixed and expanded U2OS stained with HAK-actin (color-coded with “fire” for z positions), mCLING (color-coded with “spectrum” for z positions) and tubulin (color-coded with “fire” or “spectrum” for z positions). All the bottom pictures are a combination of 2 channels. Scale bars: 10 µm

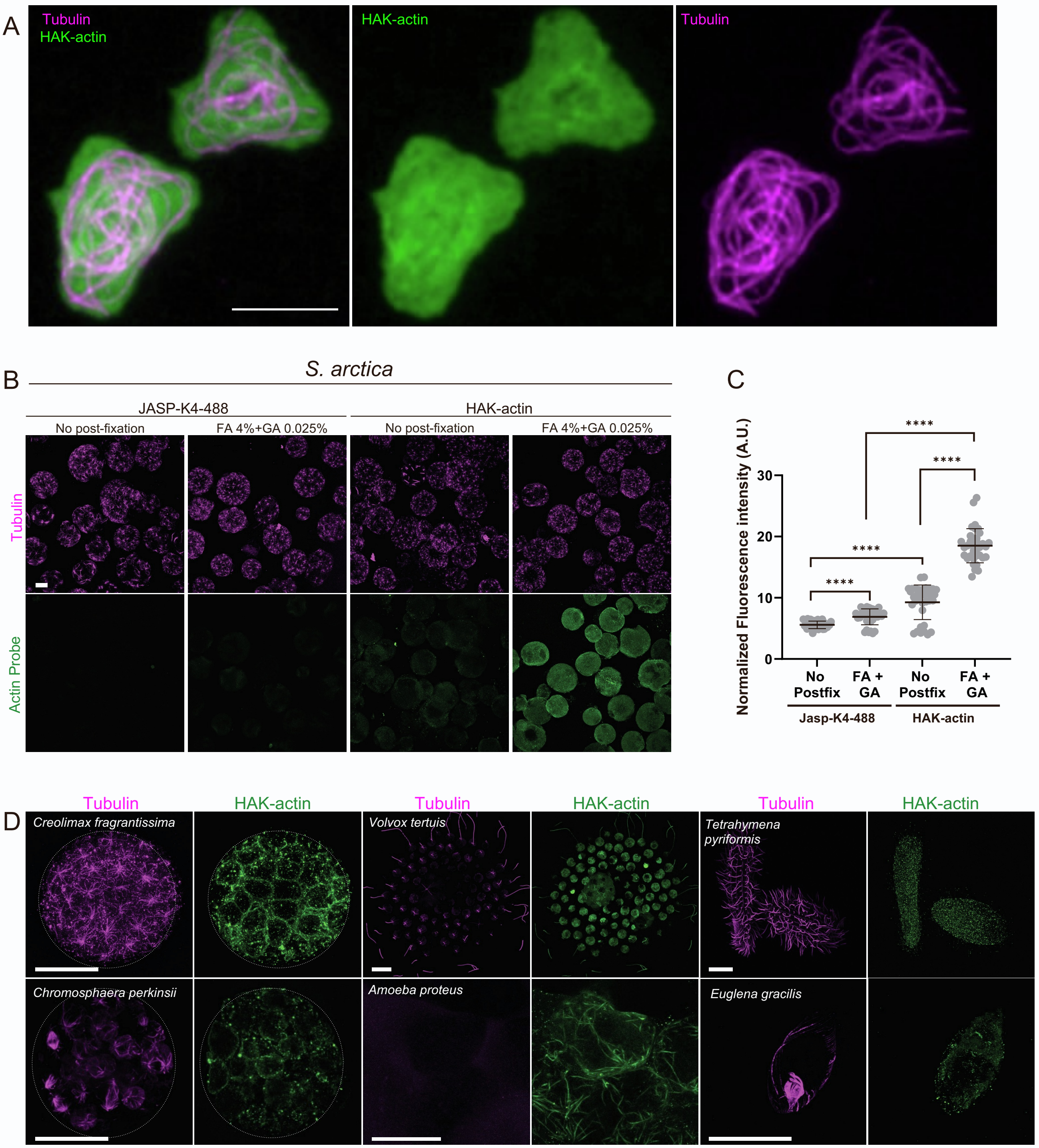


**Supplementary Figure 4. HAK-actin in platelets and microbial eucaryotes, related to Figures 3 and 4.** **(A)** Widefield image of non-expanded platelets stained for HAK-actin (green) and tubulin (magenta) Scale bar: 5 µm. **(B)** U-ExM samples of *S. arctica* labeled with JASP-K4-488 or HAK-actin probes, either without or with an additional post-fixation step (4%FA + 0.025%GA) prior to the anchoring step (FA/AA). Fluorescence intensity (normalized across images) demonstrates that JASP-K4-488 does not survive the U-ExM protocol and that HAK-actin shows increased signal with the additional crosslinking step. Scale bar: 10 µm. **(C)** Box-plot quantifying the fluorescence intensities in (B). N = 35 cells per condition. ****$p < 0.0001$ by t- test. **(D)** U-ExM samples stained with HAK-actin (green) and tubulin (magenta) in six other microbial eukaryotes: the ichthyosporeans *Creolimax fragrantissima* and *Chromosphaera perkinsii*, the green alga *Volvox tertius*, the amoebozoan *Amoeba proteus*, the ciliate *Tetrahymena pyriformis*, and the euglenid *Euglena gracilis*. Note that actin staining is observed in most species except ciliates and euglenids, where phalloidin-stained filamentous actin was never reported. Also note the apparent absence of microtubules in *A. proteus*, which may be associated with a specific cell-cycle stage. Scale bar: 10 µm.
